# Telerehabilitation for physical disabilities and movement impairment: A service evaluation in South West England

**DOI:** 10.1111/jep.13689

**Published:** 2022-04-19

**Authors:** Sarah A. Buckingham, Kim Sein, Krithika Anil, Sara Demain, Hilary Gunn, Ray B. Jones, Bridie Kent, Angela Logan, Jonathan Marsden, E D. Playford, Jenny Freeman

**Affiliations:** ^1^ Peninsula Allied Health Centre, School of Health Professions University of Plymouth Plymouth UK; ^2^ School of Health Sciences University of Southampton Southampton UK; ^3^ Centre for Health Technology, School of Nursing and Midwifery University of Plymouth Plymouth UK; ^4^ School of Nursing and Midwifery University of Plymouth Plymouth UK; ^5^ Stroke Rehabilitation, Royal Devon University Healthcare NHS Foundation Trust, William Wright House, Wonford Hospital Exeter UK; ^6^ Warwick Medical School University of Warwick Coventry UK

**Keywords:** movement impairment, physical disabilities, remote consultations, telerehabilitation

## Abstract

**Rationale, Aims and Objectives:**

Telerehabilitation was used to ensure continued provision of care during the COVID‐19 pandemic, but there was a lack of guidance on how to use it safely and effectively for people with physical disabilities and movement impairment. In this service evaluation, we aimed to collate information on practitioner and patient experiences, challenges and facilitators, and examples of best practice to inform the development of an online toolkit and training package.

**Methods:**

Guided discussions were carried out with 44 practitioners, 7 patients and 2 carers from five health and social care organisations in South West England, and analysed thematically.

**Results:**

Practitioners and patients had positive experiences of telerehabilitation and were optimistic about its future use. Recognized benefits for people with physical disabilities included greater flexibility, reduced travel and fatigue, having appointments in a familiar environment and ease of involving family members. Challenges encountered were: technological (usability issues, access to technology and digital skills); difficulties seeing or hearing patients; the lack of ‘hands‐on’ care; and safety concerns. Facilitators were supported by colleagues or digital champions, and family members or carers who could assist patients during their appointments. Key themes in best practice were: person‐centred and tailored care; clear and open communication and observation and preparation and planning. Practitioners shared tips for remote physical assessments; for example, making use of patient‐reported outcomes, and asking patients to wear bright and contrasting coloured clothing to make it easier to see movement.

**Conclusion:**

Telerehabilitation holds promise in health and social care, but it is necessary to share good practice to ensure it is safe, effective and accessible. We collated information and recommendations that informed the content of the Telerehab Toolkit (https://www.plymouth.ac.uk/research/telerehab), a practical resource for practitioners, patients and carers, with a focus on remote assessment and management of physical disabilities and movement impairment.

## 1 INTRODUCTION

The COVID‐19 pandemic resulted in significant unmet needs, with rehabilitation services being the most commonly disrupted type of health service globally.^1^ In 2020, 50% of countries reported partial disruption, and an additional 12% reported complete disruption of their rehabilitation services.^1^

Telerehabilitation, the delivery of rehabilitation via information and communication technologies,^2^ was often used to attempt to mitigate the impact of the pandemic and to ensure continued provision of care.^1^ The frequency of remote healthcare appointments (video and telephone) increased dramatically in a short time. For example, in the United Kingdom (UK) primary care, remote consultations increased from being rarely used to being employed for 90% of general practitioner appointments in April 2021.^3^

This speed of implementation has resulted in a dearth of guidance and training for practitioners. Although learning needs for healthcare professionals in using video consultations have been previously identified,^4^ there was little specific published guidance, training and support on how to undertake assessments and deliver rehabilitation remotely for people with physical disabilities.^5^ Professional bodies and clinical networks have highlighted the marked variations in approaches to telerehabilitation between and even within organisations, expressing concerns about potential inequity and inefficiency.^6^, ^7^, ^8^ The findings of our national survey of UK health and social care practitioners confirm these issues, with few practitioners receiving formal training and many reporting perceived low competence and confidence in carrying out remote physical assessments.^9^

Practitioners need to improve their knowledge and understanding of telerehabilitation for people with physical disabilities and movement impairment. In the Telerehabilitation Project,^10^ we aimed to collate information on practitioner and patient experiences, challenges and facilitators, and best practice in telerehabilitation. The overarching aim of the project was to inform an online toolkit and training package (the Telerehab Toolkit) to assist the current and future health and social care workforce in conducting safe and effective remote physical assessments and consultations.

This service evaluation had three objectives:

1. Examine the context of telerehabilitation including its use and impacts, and patient and practitioner experiences and satisfaction with the remote consultation process.
2. Explore challenges and facilitators in undertaking remote physical assessments, including how challenges have been overcome.
3. Identify examples of feasible and effective practice in telerehabilitation for people with physical disabilities and movement impairment.

## 2 METHODS

### 2.1 Governance approvals

This service evaluation was undertaken in line with Good Clinical Practice guidelines and the guidance and regulation set out by the General Data Protection Regulation.^11^ Governance approvals were obtained from each of the four participating NHS Trusts and one independent social enterprise in South West England in October 2020.

### 2.2 Overview of study design and theoretical basis

Implementation science is key to understanding change and implementing new resources, interventions or services in healthcare.^12^ The Knowledge to Action (KTA) framework,^13^ which seeks to identify and synthesise current knowledge about a particular issue and refine and implement this knowledge in an iterative process,^13^ provided the conceptual framework. The two main phases of KTA are knowledge creation and action.^13^ The service evaluation described in this paper was one source of knowledge creation (i.e., gathering information to inform the content of the toolkit).

This was a pragmatic service evaluation rather than an in‐depth qualitative study, but to ensure transparency and rigour in reporting our data collection and analysis, the Consolidated Criteria for Reporting Qualitative Research (COREQ)^14^ were followed.

### 2.3 Participants and recruitment

Purposive maximal variation sampling was used. We sought to gain the views of a range of practitioners from different occupations and with varying levels of experience of telerehabilitation, and of patients with a range of conditions (including respiratory, neurological, musculoskeletal and post‐COVID). Practitioners and patients needed to have some experience of telephone or video consultations involving assessment of physical capacity or movement; this was the only inclusion criterion.

Sampling took place through contact with lead practitioners at the five participating organisations; these practitioners were informed of the sampling criteria and they provided the names of relevant practitioners. These individuals were then contacted by the researchers (Sarah A. Buckingham or Kim Sein) via telephone or e‐mail to explain the purpose of the service evaluation and gain verbal consent to participate. For patients and carers, practitioners made initial contacts on our behalf, minimising the amount of personal data handled by the project team. If they subsequently agreed to take part, the researchers followed up via telephone or e‐mail to answer any remaining queries and schedule the discussion. With the exception of the lead practitioners, all participating practitioners, patients and carers were unknown to the researchers before recruitment commences.

### 2.4 Data collection

Data collection took place between October 2020 and April 2021. Guided discussions (individual and group) were carried out with practitioners, patients and carers. Discussions were led by male (Kim Sein) and female (Sarah A. Buckingham) post‐doctoral researchers with experience in qualitative interviews and focus groups.

Discussion points (see Tables 1 and 2) were based on knowledge gaps identified in the literature including our scoping review,^5^ findings of our survey of UK rehabilitation practitioners,^9^ and pragmatic aims of the service evaluation (i.e., what would inform the Toolkit resource). These points were reviewed by the wider research team including a Patient and Public Involvement representative.

**Table 1:** Discussion guide for practitioners

Topic for discussion	Potential prompts/areas to explore
Context and experiences	Experiences of using telerehabilitation with people with physical disabilities and movement impairment and/or people recovering from COVID‐19; how remote methods have been used; impact on service delivery
Satisfaction with the remote consultation process	Perceived benefits of remote consultations for practitioners and patients; practitioner satisfaction; perceptions of patient satisfaction
Knowledge of telerehabilitation	Sources of information used; knowledge gaps (personal or systemic); information and learning needs
Competence and confidence	Perceived competence and confidence in the delivery of telerehabilitation and remote physical assessments
Challenges and facilitators in remote consultations/physical assessments	Sources of support to practitioners and patients; environmental and practical challenges and facilitators; how challenges have been overcome
Comparison of remote and face‐to‐face appointments	Comparative perceived advantages and disadvantages; appropriate situations for different consultation modes
Optimism and intentions	Feelings about the use of remote consultations in the future; intentions to continue to use telerehabilitation
Recommendations/top tips	For carrying out remote assessments with people with physical disabilities and movement impairment

*Note*: This is not an exhaustive list and discussions covered a range of aspects of telerehabilitation.

**Table 2:** Discussion guide for patients and carers

Topic for discussion	Potential prompts/areas to explore
Context and experiences	Experiences of telephone and video appointments; what remote appointments have been used for; practitioners consulted with
Satisfaction with the remote consultation process	Patient and carer satisfaction; perceived benefits of remote appointments; patient–practitioner relationship
Knowledge of remote appointments	Sources of information used; knowledge gaps; information and learning needs
Competence and confidence	Perceived competence and confidence in remote appointments and using technology
Challenges and facilitators in remote consultations/physical assessments	Support from family members and carers; environmental and practical challenges and facilitators; how challenges have been overcome
Comparison of remote and face‐to‐face appointments	Comparative perceived advantages and disadvantages; appropriate situations for different consultation modes
Optimism and intentions	Feelings about the use of remote consultations in the future; intentions to continue to use telerehabilitation
Recommendations/top tips	For remote appointments for people with physical disabilities and movement impairment (for practitioners and patients)

*Note*: This is not an exhaustive list and discussions covered a range of aspects of telerehabilitation.

The discussions began with introductions, with researchers reminding participants of the project aims and context of the work. The researchers took care to ensure that they retained a neutral stance regarding telerehabilitation and did not express their own views. Although the discussion points were used as a guide, discussions were flexible and open‐ended rather than structured or exhaustive, and followed the points that participants wished to discuss.

In light of COVID‐19 pandemic restrictions, all discussions were carried out remotely; either online (e.g., Zoom^15^) or via telephone. Discussions took place in private locations including the researchers’ home offices, patients’ homes, and practitioners’ homes or a private workplace setting. Having telephone as an option avoided potential digital exclusion and maximized reach and representativeness of the participants.

Detailed field notes were made during the discussions. To ensure a reliable understanding of the discussed content, verbal summaries were given by researchers during and at the end of the discussion, and confirmed with participants. With explicit approval from each participant, discussions were temporarily recorded to ensure completeness and accuracy of the field notes, and to enable the capture of illustrative quotes. The audio and video recordings were deleted within 2 h of the discussion. Field notes were added after the discussions to include reflections on context and any potential preconceptions or biases of the researchers.

Data collection continued until the maximal variation sampling criteria had been met and saturation had been reached in discussions with practitioners (i.e., no new themes had been identified). For patients and carers, data collection continued until the broad sampling criteria were met.

### 2.5 Data analysis

Three researchers (Sarah A. Buckingham, Kim Sein and Krithika Anil) carried out a qualitative thematic analysis based on the guidance of Braun et al.^16^ Following familiarisation with the discussion field notes, the researchers independently^17^ coded and organized the field note data into key themes using NVivo.^17^ A pragmatic approach was taken which facilitated both deductive and inductive analysis. Deductive analysis was directed by the discussion guide and planned sections of the Toolkit, such as top tips for carrying out remote physical assessments. Inductive analysis was based on common themes arising from the data, for example, the importance of person‐centred care in telerehabilitation. The identified themes were discussed and agreed on by the researchers; there was high consensus between the researchers’ coding, with minor discrepancies resolved through further discussion. A final coding frame was developed which included illustrative quotes from participants. Although participants were not asked to provide feedback on the results of analysis, other methods were used to ensure validity and reliability including critical reflection, meticulous record‐keeping and investigator triangulation.^18^

## 3 RESULTS

### 3.1 Overview of discussions, participants and themes

Seven patients, 2 carers and 21 practitioners took part in individual discussions. In addition, four group discussions were held with 23 practitioners. Individual and group discussions were approximately 30 min to 1 h in duration. The characteristics of the 53 participants are shown in Table 3.

**Table 3:** Characteristics of participants

Practitioners: Individual discussions	*n* = 21
**Gender**	
Male	8
Female	13
**Occupation**	
Physician	4
Nurse	1
Occupational therapist	4
Physiotherapist	10
Podiatrist	1
Social worker	1
**Specialty**	
Community rehabilitation	5
General practice	1
Musculoskeletal/rheumatology	3
Neurological, stroke and/or MS	9
Pain management	1
Respiratory	2

**Practitioners: Group discussions**	***n*** = **23**
**Gender**	
Male	1
Female	22
**Occupation**	
Dietician	23
**Setting of service**	
Inpatient	3
Outpatient and community	7
Work across settings	7
Unknown	6

**Patients**	***n*** = **7**
**Gender**	
Male	4
Female	3
**Condition**	
Cystic fibrosis	1
Multiple sclerosis	1
Musculoskeletal (low back pain post‐surgery)	1
Parkinson's disease	1
Post‐COVID	1
Stroke	2

**Carers**	***n*** = **2**
**Gender**	
Male	0
Female	2
**Condition**	
Carer for patient with Parkinson's	1
Carer for post‐COVID patient	1
	**Total *n*** = **53**

Findings were classified into four overarching themes, each divided into sub‐themes: context and experiences of telerehabilitation; challenges and facilitators; practitioner recommendations for successful telerehabilitation and top tips for remote physical assessments.

### 3.2 Context and experiences of telerehabilitation

Practitioners and patients shared their experiences, views and perceptions of telerehabilitation. These were categorized into four themes described below.

i) **Adapting to remote consultations**
  Most practitioners had begun using video and telephone consultations because of the COVID‐19 pandemic; for many, this had been the only way they had been able to maintain contact with patients. Practitioners adapted rapidly to the new way of working, but at first found it to be a *‘baptism of fire’* and reported being *‘hurled into it’*. Few practitioners reported having received any formal training in conducting remote consultations or video consultation software, with the vast majority learning through experience. Practitioners recognized a need for improved training and guidance:
*‘I didn't receive any training and learned through experience’*.
  (Physiotherapist, Neurology)
*‘Everyone is still getting training on COVID, but not the subtleties of how to see and talk to patients remotely’*.
  (Physician, General Practice)
  Some practitioners talked about a need for standardised protocols and greater consistency between, and within, services. For example, several wished for a universal video consultation software program that could be used across health and social services.
  The increased use of and familiarity with remote consultations over time led to increasing satisfaction for both practitioners and patients:
*‘It took time to adapt and get used to video conferencing… clinicians are used to face‐to‐face. We became more comfortable [with it] over time’*.
  (Physiotherapist, Community Rehabilitation)
*‘Video appointments took a bit of getting used to… they got easier over time’*.
  (Patient with low back pain)
ii) **Perceived benefits for people with physical disabilities**
  Practitioners and patients reported specific benefits of remote consultations for people with physical disabilities. These included increased flexibility regarding appointment times, reduced need to travel and consequently lower stress and fatigue for patients:
*‘Remote physiotherapy can have real benefits, where the effort for the patient to attend the appointment means they may not be able to do as much at the appointment as they could if they were using video or Zoom. You are ahead of the game, able to do any exercises or movement without already being tired’*.
  (Patient with Multiple Sclerosis)
  Patients appreciated having the appointment in a familiar environment, and practitioners recognized value in observing patients in their own home as they could see how they managed their condition within their home environment. Both groups talked about video appointments making it easier to involve family members and carers. Patients were also grateful for the opportunity to access care during the pandemic:
*‘Without video appointments I would have had to wait for eight months to be seen and there could have been permanent damage’*.
  (Person with stroke)
iii) **Video versus telephone consultations**
  Video consultations were generally seen by practitioners and patients as better than telephone appointments for building rapport and carrying out physical assessments. However, telephone was useful in cases where the patient had struggled with the technology or where the practitioner wanted to quickly review progress or offer reassurance. The utility of being able to offer support and reassurance remotely, particularly in the early days of the pandemic, was often viewed by practitioners and patients as the most positive aspect of the remote consultation.
*‘It was the reassurance of being able to speak to the physio when face‐to‐face contact was not possible, that's what kept me going’*.
  (Person with stroke)
  Practitioners and patients shared the perception that telephone or video consultations might be more effective after an initial face‐to‐face assessment. For example:
*‘An initial face‐to‐face appointment might be better to build rapport’*.
  (Patient with Multiple Sclerosis)
iv) **The future of telerehabilitation**

Practitioners perceived remote consultations as a *‘useful adjunct’* to face‐to‐face care, and an important part of the whole package of care. There was a recognition that for certain individuals (such as very elderly people who are unable to access technology, or people with severe cognitive or communication impairment), face‐to‐face care remains the most appropriate option. Similarly, remote consultations may be less suitable (or not possible) where complex physical assessments or manual therapies are necessary. For these reasons, there was a strong belief that telephone and video assessments should never replace in‐person care entirely:

‘*Although telerehabilitation is a useful tool, it can never replace face‐to‐face consultations’*.

(Physiotherapist, Neurology)

Overall, there was optimism about remote consultations; practitioners and patients thought that they should and would be used more in the future:

‘*Having seen how much we can do remotely, I think remote consultations will be used a lot more now. It's here to stay I think’*.

(Physician, General Practice)

‘*Remote appointments could be used more and should be used more’*.

(Post‐COVID patient)

### 3.3 Challenges and facilitators

There were four categories of challenges in telerehabilitation: technological; difficulties seeing or hearing patients; the lack of ‘hands‐on’ care; and safety concerns. These challenges (and how they were overcome) are described below.

i) **Technological challenges**
  Technology was a commonly encountered challenge for practitioners, and even those who were confident technology users initially struggled with learning new software. Hardware problems were also common, for example, microphones not working, cameras being low quality, and unstable internet connections. Some practitioners were unable to access the technology they needed from their workplace or home, which delayed or prevented the use of video consultations. These issues tended to improve over time as workarounds were found for hardware problems and people became more familiar with specific software. Support from colleagues and informal sharing of good practice was important for many. The use of digital champions to lead on telerehabilitation and support other staff was also recommended:
  ‘*We had digital champions, therapists who felt confident with technology and were interested in it. It's been quite handy in terms of redeploying people who aren't able to do face‐to‐face… who aren't able to do their usual day‐to‐day job’*.
  (Physiotherapist, Community Rehabilitation)
  Technology issues were the challenge most frequently mentioned by patients. For patients who lacked digital skills or access to technology, support from family members was of vital importance. Two of the seven patients reported that they would not have attempted a remote appointment without the support of their families.
  ‘*I relied on support from my son to set up Zoom and make sure the appointment went smoothly. [My son] was also there for support regarding safety – I would have felt unconfident walking or moving around without him’*.
  (Person with stroke)
  Practitioners and patients felt strongly that people who lacked technology skills or access and who did not have the support of family members should not be ‘*digitally excluded’*. Recommendations included offering alternative consultation modes (telephone and face‐to‐face) and provision of digital skills training for people in need:
  ‘*Local authorities and charities need to step in… to provide technical support for people… sessions to learn how to access technology. It has to be one‐to‐one’*.
  (Social Worker, Community Rehabilitation)
ii) **Difficulties seeing or hearing patients**
  Closely associated with technological issues were difficulties with seeing or hearing patients. Practitioners felt that this impacted on the quality of the consultation. For physical assessments, the camera position was of central importance. This issue was overcome by ‘*trial and error’*, the use of a carefully placed mobile device (e.g., a tablet or laptop) rather than a desktop computer, clear instructions to patients, and help from family members or carers in positioning and moving the camera where possible.
iii) **Lack of ‘hands‐on’ care**
  The lack of ‘hands‐on’ care and ability to handle a limb was a concern for some practitioners. This was more of an issue for certain types of consultations. For example, practitioners recognized that it was not possible to adjust prostheses and orthoses nor undertake detailed objective physical assessments (such as dermatomal testing or specific muscle strength testing) remotely. The accuracy of some measures was also an area of concern, for example, arm circumference measurements, measures of balance, and timed walking tests.
  Where possible, these issues were managed by using patient‐reported outcome measures, involvement of family members or carers who could assist with physical examinations (e.g., helping to move limbs or put on and take off orthotics), and seeing patients in person as soon as practicable. Some practitioners believed that patients needed to feel reassured by a physical examination, and might have the perception that telerehabilitation is *‘second best’* or a *‘stop gap’* before they can be seen in person.
*‘Patient perception is that objective assessment (such as feeling joints) is the most important thing – but for us, history tells us a lot more. This is a difficulty with telerehab’*.
  (Physiotherapist, Musculoskeletal)
*‘Patients want a hands‐on appointment to feel listened to. They feel vindicated. Remote doesn't seem to always provide that reassurance’*.
  (Physiotherapist, Respiratory)
  However, this view was not supported by the patient discussions in this service evaluation. Rather, it was *‘encouragement and reassurance’, and ‘motivation and emotional support’* that patients felt they needed most and speaking to practitioners remotely met these needs.
iv) **Safety concerns**
  Many practitioners expressed some concerns over patient safety in telerehabilitation, particularly when the patient was alone. This sometimes led to them being risk averse and avoidant in conducting remote physical assessments or asking patients to exercise:
*‘I probably hold back more via video… I tend to be more risk averse*’.
  (Physiotherapist, Neurology)
*‘I am hesitant about looking at any physical oriented assessments, such as sit‐to‐stand, because of risk*’ [with reference to patients with cancer or the elderly]
  (Dietician, Outpatient and Community)
  Practitioners talked about the importance of carrying out a thorough risk assessment, including weighing up the benefits and risks of different consultation modes (including the risk of taking no action), and considering patients’ health status and surroundings. They recognized that clinical judgement was essential, but felt that safety guidance provided by their organisation should be clear. Support from family members and carers was highly valued, and commonly used to check for environmental safety hazards and provide standby assistance to optimize safety during physical activities (such as assessment of mobility and balance).

### 3.4 Practitioner recommendations for successful telerehabilitation

Practitioners made some recommendations for carrying out telerehabilitation successfully. The key themes were: person‐centred care; communication and observation; and preparation and planning.

i) **Person‐centred care**
  Person‐centred care was seen as a cornerstone of telephone and video consultations. The importance of patient preference was frequently mentioned. Practitioners believed that, with their guidance and support, patients should be able to make informed choices regarding their care, including the most suitable type of consultation (telephone, video or face‐to‐face). Both practitioners and patients felt that remote consultations enabled personalized rehabilitation that could be tailored to the individual's needs:
  ‘*It is important to listen to what patients want and need. Remote work makes this model easier*’.
  (Physiotherapist, Respiratory)
  ‘*It's about you*’.
  (Patient with Multiple Sclerosis)
  Practitioners reported that a positive consequence of person‐centred, remote care was that it appeared to foster more independence by patients to manage their own health:
  ‘*Telerehabilitation is helping to move patients from passive recipients of care to active self‐managers*’.
  (Physiotherapist, Neurology)
ii) **Communication and observation**
  Although communication and observation are crucial skills of health and social care practitioners, these were seen as even more vital in remote rehabilitation. Some difficulties with communication may be experienced, for example, not being able to see or hear patients clearly. Many practitioners found reading body language and non‐verbal signs more difficult via video consultation. They recognized the need to carefully observe the patient and their environment, and to use ‘clues’ based on what the patient says and does to inform diagnosis and treatment. Clear and open communication was seen as particularly important; practitioners recommended using simple instructions, summaries and repeating back, and openly discussing observations with the patient. Practitioners highlighted that the clinical decisions they make often need to become an explicit conversation during remote consultations, where they may have been an implicit observation in face‐to‐face appointments.
  ‘*[In a remote consultation] you rely more on communication and conversation skills. I try to be overt with the patient about the risks and benefits of different options… make the implicit explicit… to encourage shared decision‐making*’.
  (Physician, Neurology)
iii) **Preparation and planning**
  The need for preparation and planning was emphasized not only by practitioners, but also by patients and family members. Most practitioners had learned through experience that telerehabilitation did not always save time:
  ‘*We didn't realize it at first… [but] you need to allow at least the same amount of time for virtual and face‐to‐face appointments*’.
  (Physiotherapist, Musculoskeletal)
  Practitioners recognized that while the need for travel was removed in a video consultation, it was necessary to allow for ‘digital travel time’. This included time spent dealing with technical issues, and repositioning the camera and/or the patient to enable effective remote physical assessment. As in a face‐to‐face consultation, the need to allow for time between appointments for documentation and reflection was highlighted. Practitioners generally found conducting screen‐based assessments more tiring than face‐to‐face assessments, reporting the need for scheduled breaks to reduce fatigue.
  One of the ‘top tips’ given by patients was to practise using the video consultation software in advance, to improve familiarity and confidence and reduce the time spent solving technical issues during the scheduled appointment. Several practitioners reported sending information to patients in advance, more frequently than they would have done before a face‐to‐face appointment; this included instructions, self‐report questionnaires, examples of exercises and links to web‐based resources.

### 3.5 Top tips for remote physical assessments

Practitioners provided specific, practical recommendations for carrying out remote physical assessments. Some examples of these are included in Table 4.

**Table 4:** Recommendations made by the practitioners for carrying out remote physical assessments

Recommendation	Rationale
Use telephone triage	Telephone triage was a useful tool for assessing the patient's background, medical and medication history and deciding on the best method for follow‐up treatment and management
Make use of patient‐reported outcomes	Patient‐reported outcomes (e.g., validated questionnaires for pain, fatigue, mobility and quality of life) completed in advance proved useful in informing the consultation and related discussions.
Find a suitable space	Considering the environment in which the consultation was undertaken was important, both for the practitioner and patient. This included ensuring confidentiality, and consent for involvement of others such as family members. Ensuring the room was well lit and without glare allowed better observation and communication.
Ask the patient to wear bright and contrasting colours of clothing	Bright and contrasting coloured clothing (e.g. different coloured trousers, socks and shoes) helped practitioners to see movement and distinguish between body regions.
Involve family members and carers	Family members or carers, when available, proved invaluable in providing physical assistance during assessments, and helped with using technology or moving the camera to enable a more effective assessment to be undertaken.
Safety is paramount, but avoid risk aversion	Practitioners recognized that safety is essential but that there is a need to try not to be too risk averse in physical assessments. Completion of a risk assessment, clinical judgement, and making use of family members and environmental supports to optimize safety were all key considerations. There was widespread recognition that a face‐to‐face appointment should be employed where a safe and effective remote assessment cannot be completed.

## 4 DISCUSSION

In this service evaluation, COVID‐19 was clearly a major catalyst in increasing the use of telerehabilitation. Overall, practitioners and patients were optimistic about using remote consultations, and felt that they should be used to complement, rather than replace, face‐to‐face care (i.e., as part of a ‘hybrid’ approach). Advantages for people with physical disabilities were recognized, including greater flexibility, reduced travel, lower stress and fatigue, having appointments in comfortable and familiar environments, and ease of involving family or carers. Technical and practical challenges, although encountered, were usually overcome with practice, learning through experience and support from colleagues (practitioners) and family members (patients). However, practitioners expressed a need for training, guidance and standardised protocols for carrying out remote physical assessments safely and effectively.

The findings support and add to those of previous studies. The perceived feasibility and acceptability of telerehabilitation for practitioners and patients have been reported in various health and social care settings. In a US survey conducted during the COVID‐19 pandemic, patients experiencing virtual physical, occupational and speech and language therapy reported numerous benefits of remote therapy and high levels of satisfaction with their care.^19^ Positive experiences of virtual outpatient clinics have been reported by patients in the UK,^20^ Australia^21^ and Israel.^22^ Our survey of UK rehabilitation practitioners found generally positive perceptions and satisfaction with telerehabilitation.^9^ The finding that practitioner satisfaction with telerehabilitation increased with greater familiarity and experience has been reported elsewhere. For example, Lawford et al.^23^ noted a shift in perceptions of physiotherapists who were prescribed exercise therapy for osteoarthritis via telephone, after seeing positive outcomes for patients.^23^

A key finding of this service evaluation is the perception by practitioners and patients that telerehabilitation is a useful tool that should not replace face‐to‐face therapy, but should be part of the wider package of care. There were suggestions that telephone and video‐based consultations may be more appropriate and effective after an initial face‐to‐face appointment; this aligns with previous qualitative research where clinicians and patients felt that an in‐person consultation before meeting remotely provided a strong basis for a good clinical relationship.^24^

Service evaluation participants emphasised the need to reduce digital exclusion as far as possible; this can be achieved by offering alternatives to video‐based appointments (i.e., telephone and face‐to‐face) and signposting to digital skills training. Digital inclusion has been recognized as an essential component for planning and evaluating remote consultation services in a new framework developed by Greenhalgh et al.^24^

The challenges of telerehabilitation identified in this service evaluation were in line with those recognized in our survey^9^ and previous studies.^5^ The challenges most frequently discussed by practitioners and patients were: technical issues (usability problems with hardware and software, difficulties accessing technology and a lack of digital skills); difficulty seeing or hearing patients; concerns about the lack of ‘hands‐on’ care; and safety concerns. Technical issues, experienced by both practitioners and patients, are a frequently reported barrier to telerehabilitation.^20^, ^22^, ^25^, ^26^ Problems with seeing or hearing patients, and practitioners’ concerns about the lack of ‘hands‐on’ care have also been reported previously.^27^, ^28^, ^29^ Many practitioners had safety concerns, particularly when patients were alone; this led to risk aversion when conducting remote physical assessments. The implications of this risk aversion, and how this may impact on clinical practice and patient care, should be carefully considered. Practitioners wanted clear guidance and training on conducting risk assessments remotely.

The main facilitators for practitioners were supported from colleagues who could share their own experiences and provide informal guidance, and digital champions (where available) to provide technical support. A key facilitator for patients was support from family members or carers. The importance of involving carers in telerehabilitation has been emphasised previously,^25^, ^30^ but our findings show how they can specifically support the remote rehabilitation process for people with physical disabilities. These included: helping to set up or use the technology; positioning and moving the camera; providing ‘hands‐on’ assistance (e.g., moving or supporting limbs and helping to put on and take off orthotics); checking the environment for safety hazards and providing standby assistance during physical assessments.

A strength of this service evaluation is the exploration of similarities and differences in practitioner and patient perspectives of telerehabilitation. There was general satisfaction from both groups with the telerehabilitation process, and agreement regarding the perceived benefits and challenges encountered. The main difference was in relation to the lack of ‘hands‐on’ care; some practitioners believed that patients would miss the physical aspect of care, and that this would be necessary for reassurance. However, this was not mentioned in the patient discussions; patients were satisfied with the rehabilitation they had received via video or telephone and talked about the ‘encouragement and reassurance’ which had met their needs. While practitioners and patients recognized that it is not possible to do everything remotely, it would be beneficial to further explore perceptions of the necessity of physical touch in rehabilitation. It would also be interesting to examine whether these views change after the pandemic; was it that encouragement and reassurance were a key priority at a time when many people were experiencing social isolation, and when hands‐on therapy was less readily accessible?

An important output of this study is the sharing of recommendations for best practice in telerehabilitation. The three overarching themes were: the provision of person‐centred, tailored rehabilitation; the necessity of clear, explicit communication and observation between practitioners and patients; and preparation and planning by practitioners and patients. Many of the recommendations and skills gained from telerehabilitation could also be applied to enhance face‐to‐face rehabilitation. Practitioners also shared specific, practical tips for carrying out remote physical assessments.

The findings from this service evaluation have been collated and triangulated with our scoping review^5^ and survey^9^ to inform the content of the Telerehab Toolkit (www.plymouth.ac.uk/research/telerehab).^10^ The Toolkit was developed iteratively using the KTA framework,^13^ and provides information and guidance for practitioners and patients, with a focus on telerehabilitation for people with physical disabilities and movement impairment. It is apparent from our findings, and the lack of existing guidance and protocols on remote physical assessments,^5^ that this is a much needed resource. Most practitioners felt they lacked training on adapting consultations to a remote setting and wanted guidance on various aspects of telerehabilitation including effective communication, overcoming technical problems, information governance and safety considerations.

Based on our findings, we propose the following for telerehabilitation practice and policy in health and social care:

- **Education and guidance**
  o Education, training and upskilling of practitioners in various aspects of telerehabilitation—including digital skills, practical and communication skills, involving caregivers, information governance and safety.
  o Standardised guidance and protocols (which can be adapted to local contexts), including: how to prepare for telephone and video‐based consultations; decision aids for when to use remote versus face‐to‐face consultation modes; information governance; and conducting risk assessments.
- **Use of standardised software for video‐based consultations**
  o Where possible, the software used should be consistent across health and social care organisations. Clear guidance and instructions on use should be available for practitioners and patients.
- **Technical support for practitioners and patients**
  o Use of digital champions within organisations to lead on telerehabilitation and provide technical support to practitioners.
  o Signposting to and/or provision of digital skills training for patients.
- **Person‐centred, tailored telerehabilitation**
  o Care provided through telerehabilitation should be person‐centred and tailored to the needs and preferences of the individual. Practitioners should aim to support self‐management.
- **Clear communication and observation**
  o Clear and open communication between practitioners, patients and carers from preparation for the appointment through to virtual assessment and follow‐up.
  o Careful observation of the patient in their home environment and use of ‘clues’ to aid diagnosis and treatment, with shared decision‐making between the patient and practitioner.
- **Preparation and planning**
  o Careful preparation and planning of telephone and video‐based consultations by the practitioner and patient. Information may be sent to patients in advance of the appointment (e.g., examples of exercises and useful web links).
  o Adequate time should be allowed for remote consultations—including ‘digital travel time’ for dealing with technical problems, documentation and reflection between cases.
- **Telerehabilitation should complement face‐to‐face care**
  o Telerehabilitation is only one tool in the whole care package. It is not suitable for every person or occasion. The most appropriate consultation mode should be selected based on clinical judgement and patient preference where possible. Telephone and face‐to‐face appointments should be offered as an option, in particular where there is a risk of digital exclusion.

### 4.1 Strengths and limitations

The main strengths of this service evaluation were the representation of a range of practitioners in five different health and social care organisations, consideration of the patient's perspective, and the novel focus on the movement‐related aspects of telerehabilitation.

Some limitations should be considered. First, the service evaluation was conducted in one region, South West England, which may limit generalisability. Although the COVID‐19 pandemic was experienced universally, the impact on services and associated experiences may vary in different settings or contexts. Nevertheless, we believe that the findings are of relevance and practical use to practitioners and patients, in addition to service providers considering implementing or expanding their telerehabilitation services. Second, whilst the individual discussions were held with a wide range of health and social care practitioners, the group discussions included only dieticians. This was the most pragmatic format due to the relatively large number of dieticians and for reasons of availability. Third, all of the participants in this service evaluation had experience of remote consultations involving physical assessments, and none held negative views of telerehabilitation. The views of practitioners and patients who are more reluctant to use telerehabilitation should be explored, to identify further barriers and how they may be overcome. Finally, although the sampling criteria for patients were met, with representation of patients with a range of conditions, the numbers recruited were small. Recruitment during the COVID‐19 pandemic presented practical challenges; with the work conducted when pressures on health services were high, practitioners were experiencing conflicting priorities with limited time and resources for involvement in research and evaluation. This meant that we cannot be confident that data saturation was reached with regard to patient perspectives. In addition, only two carers took part in the evaluation; as it was clear from the discussions with patients and practitioners that family members and carers play a vital role in telerehabilitation, future studies should explore the perspectives and needs of carers in more depth.

## 5 CONCLUSION

Health and social care practitioners rapidly adopted telerehabilitation in response to the COVID‐19 pandemic. In this service evaluation, we found that practitioners and patients had generally positive experiences of rehabilitation delivered via video and telephone, and were optimistic about its future use in a hybrid approach combined with face‐to‐face care. However, some challenges still need to be addressed to ensure it is safe, effective and accessible to a wide range of patients. We identified information needs and collated examples of best practice and recommendations to inform the content of the Telerehab Toolkit, an online resource for practitioners and patients, with a focus on the remote assessment and management of physical disabilities and movement impairment.

## AUTHOR CONTRIBUTIONS

Jenny Freeman is the Principal Investigator and conceived the project. All authors contributed to the service evaluation design. Sarah A. Buckingham and Kim Sein carried out recruitment, data collection and analysis, and Krithika Anil assisted with data analysis. Sarah A. Buckingham wrote the first draft of the manuscript; all authors reviewed and edited subsequent versions and approved the final version.

## CONFLICTS OF INTEREST

The authors declare no conflicts of interest.

## Data Availability

Requests for data sharing should be made to the Principal Investigator (PI; J Freeman) in the first instance. Requesters will be asked to complete an application form detailing specific requirements, rationale, and proposed usage. Requests will be reviewed by the PI, who will consider the viability and suitability of the request and the credentials of the requester. Where access to requested data is granted, requesters will be asked to sign a data sharing agreement. Requested data will be made available, along with supporting documentation (eg, data dictionary) on a secure server or through other secure data transfer method.
